# The Serotonergic System and Bone Metabolism During Pregnancy and Lactation and the Implications of SSRI Use on the Maternal-Offspring Dyad

**DOI:** 10.1007/s10911-023-09535-z

**Published:** 2023-04-22

**Authors:** Hannah P Fricke, Laura L Hernandez

**Affiliations:** Animal and Dairy Sciences Department, University of Wisconsin-Madison, Madison, WI USA

**Keywords:** Lactation, serotonin, SSRI, bone, calcium

## Abstract

Lactation is a physiological adaptation of the class Mammalia and is a product of over 200 million years of evolution. During lactation, the mammary gland orchestrates bone metabolism via serotonin signaling in order to provide sufficient calcium for the offspring in milk. The role of serotonin in bone remodeling was first discovered over two decades ago, and the interplay between serotonin, lactation, and bone metabolism has been explored in the years following. It is estimated that postpartum depression affects 10–15% of the population, and selective serotonin reuptake inhibitors (SSRI) are often used as the first-line treatment. Studies conducted in humans, nonhuman primates, sheep, and rodents have provided evidence that there are consequences on both parent and offspring when serotonin signaling is disrupted during the peripartal period; however, the long-term consequences of disruption of serotonin signaling via SSRIs during the peripartal period on the maternal and offspring skeleton are not fully known. This review will focus on the relationship between the mammary gland, serotonin, and bone remodeling during the peripartal period and the skeletal consequences of the dysregulation of the serotonergic system in both human and animal studies.

## Introduction

There are approximately 4,600 species of vertebrates that belong to the class Mammalia. One of the hallmarks of mammals is the mammary gland and the ability to lactate, which is the product of more than 200 million years of evolution [1]. Despite the importance and prevalence of this physiological function, the evolutionary origin of lactation and the mammary gland is largely unknown; the mammary gland may have evolved from sweat glands, sebaceous glands, apocrine glands, or some combination of all three [2]. Lactation strategies and milk composition vary dramatically among mammals. Monotremes, or egg-laying mammals, do not have nipples. Instead, the mammary ducts secrete milk directly from the skin in association with hair follicles [3]. Marsupials such as the tammar wallaby can perform asynchronous concurrent lactation, in which adjacent mammary glands can produce milk of varying composition to two different joeys of different ages [4]. The largest member of the class Mammalia, the blue whale, has an estimated energy output of 4,000 MJ/d during lactation to support the growing calf, which will experience an increase of 17,000 kg, or approximately 37,500 lbs., in mass throughout the 6 to 7 month lactation period [5, 6]. Milk composition also varies between mammals based on the needs of the offspring. Human and bovine milk have similar percent fat compositions (2.10-4.00 and 3.60, respectively), while ovine milk is composed of 5.70% fat [7].

In humans, lactation is a beneficial process for both parent and offspring, providing nutritionally complete milk for the baby’s growth and development, as well as immune protection for the infant by providing antimicrobial agents, immunomodulating agents, and anti-inflammatory factors [8]. Breastfeeding is important for bonding between parent and infant [9]. The World Health Organization recommends exclusive breast milk for the infant’s first six months, highlighting the unparalleled benefits of breastfeeding to both members of the breastfeeding dyad [10]. However, there are extenuating factors to consider when determining what is best for both mother and offspring, and a prominent extenuating factor is the diagnosis and treatment of mental illness.

According to the National Institute of Mental Health, 19.4 million adults (7.8% of the population) experienced at least one major depressive episode in the United States in 2019, and the prevalence was higher among females than males (9.6% and 6.0%, respectively) [11]. Females are most vulnerable to experiencing depression during their childbearing years, and postpartum depression is estimated to affect 10 to 15% of the population [12, 13]. Selective serotonin reuptake inhibitors (SSRI) are commonly used as the first-line therapy for the treatment of depression among the general population, as well as during pregnancy and lactation [14]. Both SSRIs and lactation are independently associated with a decrease in bone mass, possibly due to the role of serotonin in bone homeostasis and lactation-induced bone remodeling [15]. When the two are combined, there is evidence that there are persistent effects on the skeletal health of the mother, as well as impacts on the bones of the offspring [16, 17]. This review will explore the role of serotonin in bone remodeling and development of both parent and offspring, the usage and significance of serotonin and SSRIs during the peripartal period, and the implications of perturbations of the serotonergic system during early development of the infant.

## Serotonin & Selective Serotonin Reuptake Inhibitors

### Serotonin

Serotonin, or 5-hydroxytryptamine (5-HT), was first discovered in 1937 by Vittorio Erspamer for its role in inducing contraction of the gut in rodents and the uterus in rats, and he named his discovery enteramine. Over a decade later, it was characterized for its vasoconstrictive action in bovine serum and called serotonin, and approximately four years later, enteramine and serotonin were found to have the same structure. [18–20]. Though it is most popularly known for its central role as a neurotransmitter, commonly dubbed the ‘happy hormone,’ serotonin plays many roles throughout the body as a hormone. Only a small percentage of serotonin is found in the brain, produced primarily by the neurons of the raphe nuclei; the rest of the body’s serotonin is found in the periphery, where 90 to 95% is produced by the enterochromaffin cells of the gut [19]. Serotonin is a biogenic monoamine derived from the amino acid tryptophan that emerged very early in evolution and is present in nearly every living organism [21]. Centrally, serotonin is responsible for regulating temperature, mood, appetite, sleep, and sexual behaviors [22]. In the periphery, serotonin is involved in the regulation of a multitude of physiological processes, including vasoconstriction, gastrointestinal motility, bone homeostasis, inflammation, and lactation [18, 23–26]. Though tryptophan and the serotonin precursor 5-hydroxytryptophan (5-HTP) can cross the blood-brain barrier, serotonin itself cannot, and so the central and peripheral pools of serotonin remain separate [27, 28].

Serotonin is derived from the amino acid L-tryptophan in two steps. First, tryptophan is hydroxylated to 5-HTP by the rate-limiting enzyme of serotonin synthesis, tryptophan hydroxylase (TPH). There are two distinct isoforms of TPH, transcribed by two separate genes: TPH1 is expressed in the periphery, while TPH2 is found centrally [29, 30]. After the first step, 5-HTP is then decarboxylated by aromatic L-amino acid decarboxylase (AADC) to form serotonin. The majority of serotonin is then metabolized by monoamine oxidase (MAO) to form 5-hydroxyindole acetaldehyde (5-HIAL), which is inactive. Aldehyde dehydrogenase further acts on 5-HIAL to form 5-hydroxyindole acetic acid (5-HIAA), which is excreted in the urine. In this way, 5-HIAA is considered an indicator of whole-body serotonin turnover [31]. Serotonin also serves as a precursor to the hormone melatonin. Metabolism of serotonin to melatonin primarily occurs in the pineal gland in a two-step reaction. First, serotonin is metabolized to N-acetylserotonin by the enzyme aralkylamine N-acetyltransferase (AANAT). Then, the enzyme hydroxyindole O-methyltransferase methylates N-acetylserotonin, forming acetyl-5-methoxytryptamine, which is more commonly known as melatonin. Serotonin levels in the pineal gland are elevated during the daytime compared to the night, while the inverse is true for melatonin, and this is correlated with AANAT activity [32–34].

Tryptophan is an essential amino acid in humans, and thus must be obtained in the diet. Synthesis of central serotonin is heavily dependent on the bioavailability of tryptophan in the plasma [35]. Nearly 95% of serotonin in the body is derived from dietary tryptophan, though it is estimated that only 1% of tryptophan is used for serotonin synthesis [36]. The remaining tryptophan is incorporated into proteins or metabolized into kynurenine, which are crucial in producing cellular energy in the form of nicotinamide adenine dinucleotide (NAD+) and regulating the immune system. Kynurenine and its metabolites are widely considered to be a mechanism in major depressive disorder. Tryptophan is metabolized into kynurenine by one of two enzymes: tryptophan-2,3-dioxygenase (TDO) or indoleamine-2,3-dioxygenase (IDO). Kynurenine can then be further broken down along two distinct pathways. One pathway produces kynurenic acid, which is an antagonist for the N-methyl-d-aspartate (NMDA) receptor and is considered neuroprotective. The other pathway produces quinolinic acid, which is an NMDA receptor agonist and is considered neurotoxic [37, 38]. Overactivation of the kynurenine pathway was first hypothesized to shift tryptophan metabolism away from serotonin synthesis, resulting in a deficiency in serotonin, but results of subsequent studies on the role of tryptophan depletion in depression have been inconclusive [39, 40].

### Serotonin Signaling

There are at least 14 different types of serotonin receptors belonging to 7 families of receptors. Apart from 5-HT_3_, which is a ligand-gated ion channel, all the receptors are G-protein coupled receptors (GPCR) [41]. The GPCR serotonin receptors found in mammals are estimated to have evolved over 750 million years ago [42]. The 5-HT_1_ receptor group comprises five receptors: 5-HT_1A_, 5-HT_1B_, 5-HT_1D_, 5-ht_1e_, and 5-HT_1F_. They are functional in a variety of tissues and are mostly linked to the GPCR G_i/o_, thus inhibiting cyclic AMP (cAMP) formation. Three receptors, 5-HT_2A_, 5-HT_2B_, 5-HT_2C_, make up the 5-HT_2_ receptor group. This group of receptors preferentially couple to the GPCR G_q/11_ and increase inositol phosphates and cytosolic calcium, emphasizing the role of 5-HT_2_ receptors in muscle contraction and brain stimulation [43]. The 5-HT_2A_ receptor is of particular interest, as it is associated with normal brain function, and its activation is associated with the stimulation of various hormones, including adrenocorticotropic hormone (ACTH), oxytocin, and prolactin [44]. The 5-HT_3_ receptors, 5-HT_3A_ and 5-HT_3B_ are found in both the periphery and the central nervous system and play a role in the gut as well (Nichols 2008). The final three serotonin receptors, 5-HT_4_, 5-HT_6_, and 5-HT_7_ all preferentially couple to the GPCR G_s_ and increase cAMP formation [43].

The serotonin transporter (SERT) is present both centrally and in the periphery, and SERT is encoded by the gene *SLC6A4*. The serotonin transporter belongs to the neurotransmitter sodium symporter (NSS) transporter family. The NSS transporter family also includes transporters for dopamine and norepinephrine [45]. In the periphery, the enterochromaffin cells of the gut synthesize serotonin and release it into blood plasma, at which point the majority of the serotonin in circulation is transported into platelet cells by SERT. Once in the platelets, serotonin is separated into dense granules by vesicular monoamine transporters or is degraded by MAO [46]. Platelets do not contain TPH and cannot synthesize serotonin; therefore, SSRI-induced blockage of SERT results in the depletion of the serotonin stored within platelets [47]. Further, plasma serotonin concentrations regulate the platelet surface expression of SERT in a biphasic manner. Increasing plasma serotonin concentrations initially result in an increase in SERT expression and therefore serotonin uptake but then decreases in response to the higher serotonin concentration [46].

### Selective Serotonin Reuptake Inhibitors

Serotonin is widely implicated in the pathology of several mood disorders. The idea of the involvement of serotonin in depression was first postulated by Schildkraut in 1965 when he proposed the ‘catecholamine hypothesis’ to explain depression [48]. Since then, serotonin has been implicated in psychiatric disorders such as depression, anxiety disorders, obsessive-compulsive disorder (OCD), and post-traumatic stress disorder (PTSD) [49]. The link between low serotonin availability and depression is evident both centrally and in the periphery. For instance, it has been shown that there is a decrease in serotonin transporter sites and platelet serotonin levels (45% and 30%, respectively) in humans with untreated depression versus their non-depressed counterparts [50].

Selective serotonin reuptake inhibitors are the most popular class of antidepressants in the United States. The medications in this class of antidepressants function as their name suggests: they target the serotonin transporter to prevent reuptake of serotonin into the pre-synaptic nerve ending, which increases the levels of serotonin in the synaptic cleft [51]. Before the introduction of SSRIs, tricyclic antidepressants (TCA) and monoamine oxidase inhibitors (MAOI) were the main classes of antidepressants and functioned by either inhibiting the uptake of monoamines or by inhibiting monoamine oxidase. Tricyclic antidepressants and MAOIs were first made available in the 1950s, though due to the many adverse side-effects seen in both classes, they are no longer are used as the first line of treatment for major depressive disorder (MDD) and are rarely prescribed today [52].

Fluoxetine hydrochloride was the first SSRI introduced in the United States under the brand name Prozac in 1987 [53]. It was first described over a decade earlier as (3-(p-trifluoromethylphenoxy)-N-methyl-3-phenylpropylamine, or Lilly 110,140, in 1974 [54]. Though it is no longer the most commonly prescribed SSRI, it remains one of the most widely used antidepressants. In 2018, fluoxetine was the 20th most commonly prescribed medication in the United States and the second most commonly prescribed antidepressant, surpassed only by sertraline (Zoloft), another member of the SSRI family of antidepressants [55]. Fluoxetine exerts its action by blocking SERT and subsequently hindering reuptake of serotonin into the presynaptic neurons, and it also has moderate activity at both the 5-HT_2A_ and 5-HT_2C_ receptors [56]. Fluoxetine also has effects on peripheral serotonin. Treatment of fluoxetine for 12 weeks in depressed patients resulted in a dramatic decrease in serotonin transporter sites and platelet serotonin content compared to both baseline values and non-depressed counterparts [50]. Unlike the other SSRIs, fluoxetine has a considerable half-life of 1–3 days, and an active metabolite, norfluoxetine, which has a half-life of 7–15 days [57]. Fluoxetine is demethylated to norfluoxetine by cytochrome p450 isozymes, primarily CYP2D6 and CYP2C9 in the liver, and is mainly excreted in urine when administered orally [58, 59]. Fluoxetine inhibits its own metabolism via the inhibition of CYP2D6, leading to its nonlinear kinetic profile [58].

## The Peripartal Period

### Serotonin and the Reproductive Cycle

Males and females differ in their reported rates of depression. According to the World Health Organization, unipolar depression is twice as common in women compared to men [60]. Gender bias in psychological disorder treatment is believed to be a contributing factor to this disparity, and social and cultural factors are believed to make women more susceptible to depression. However, it is also important to note that there may be biological factors as well. The possible role of ovarian hormones in the treatment of mental illness was first explored over a century ago, predating the discovery of serotonin [61]. The estrogen receptor has two isoforms, Erα and Erβ, which are transcribed from two separate genes [62]. Estrogen has been shown to reduce neurite growth of serotonergic cells that express Erα and Erβ, which may contribute to the sexual dimorphism of serotonergic innervation in the brain [63]. Progesterone also has a receptor that exists in two isoforms, PR-A and PR-B, both encoded by the same gene [64, 65]. In the brain, serotonin is colocalized with progestin receptors, which are present on serotonin neurons, suggesting that progesterone acts on the serotonergic system in the brain to increase serotonin synthesis [66]. Further, there is evidence that estrogen modulates serotonin receptor availability via interaction with estrogen receptors in the brain and that it may act on serotonergic neurons as well [67, 68]. Estrogen treatment in ovariectomized and hysterectomized rhesus macaques induced the expression of progestin receptors in the dorsal raphe nuclei, further underlining the dynamic interplay between female gonadal hormones and the serotonergic system in the brain [69].

Due to the relationship between serotonin and ovarian sex hormones, there is evidence that estrogen and progesterone play a role in mood. Progesterone withdrawal resulted in depression-like behavior in rodents without altering serotonin levels or serotonin metabolism centrally or peripherally, and this depression-like behavior was not remedied with fluoxetine administration [70]. In rats, daily progesterone administration over 28 days resulted in an increase in both tryptophan and serotonin in the duodenal mucosa (2.1 fold and 1.5 fold, respectively) as well as an increase of tryptophan and serotonin in serum (1.5 fold and 4.1 fold, respectively) [71]. When ovariectomized rodents were administered 17β-estradiol, a decrease in depression-like behavior was observed. However, when rodents were pretreated with tamoxifen, an estrogen receptor antagonist, the antidepressant effect of estrogen administration was no longer observed [72]. Interestingly, the efficacy of SSRIs may be dependent on estrogen. In humans, reproductive-aged women were more sensitive to fluoxetine than maprotiline, a tetracyclic antidepressant, but there was no difference in responsiveness to the antidepressants in men [73]. In rodents, females in estrus have a higher sensitivity to the antidepressant effect of fluoxetine than males [74]. Fluoxetine also affects estrogen. Administration of fluoxetine to ovariectomized rats treated with estrogen suppressed the levels of circulating estrogen [75]. One of the mechanisms of action of fluoxetine is a desensitization of the 5-HT_1A_ receptor, which has been implicated in mood disorders due to its role in modulating serotonergic signaling in the brain [76, 77]. There is evidence that estradiol accelerates the desensitization of the 5-HT_1A_ receptor induced by fluoxetine [78]. The administration of 17β-estradiol and progesterone to ovariectomized rats decreased 5-HT_1A_ receptor mRNA expression in the dorsal raphe nucleus, and estradiol benzoate treatment in ovariectomized rats co-modulated somatodendritic 5-HT_1A_ receptors in the median raphe nucleus, further highlighting the link between female gonadal steroids and the serotonergic system in the brain [68, 79].

The link between estrogen and serotonin can be easily observed in the context of postmenopausal women and hormone replacement therapy. Menopausal women have a worse response to SSRI antidepressant treatment than premenopausal women, and this difference was independent of many possible confounding factors, including age, education, employment, and whether the SSRI was prescribed for either 3 or 6 months [80]. Further, hormone therapy in postmenopausal women was correlated with greater efficacy of SSRI treatment compared to postmenopausal women that were not undergoing any type of hormone therapy [81]. Estrogen administration itself has antidepressant properties. A study performed in perimenopausal women demonstrated a full or partial therapeutic response to estrogen administration in 80% of subjects after three weeks of treatment compared to the placebo group, in which only 22% showed a therapeutic response [82]. These studies in postmenopausal populations further emphasize the relationship between estrogen and serotonin.

### Serotonin During the Peripartal Period

During the peripartal period, the mammary gland undergoes dramatic changes to prepare for lactation. These changes are modulated by both endocrine and autocrine-paracrine signals, which orchestrate mammary gland development, lactogenesis, and lactational homeostasis. There are many factors in these processes, and serotonin is a predominant one. The mammary gland contains TPH1, allowing for serotonin synthesis. The serotonergic system in the mammary gland works in an autocrine-paracrine fashion to regulate mammary gland development, lactational homeostasis, and involution [26]. Serotonin is a mediator of homeorhesis, or the orchestrated changes in metabolism that are required to support a physiological state such as lactation [83, 84] There is a distinct interaction between serotonin and prolactin, a polypeptide hormone that is crucial in mammary gland development and lactation. Prolactin production is most commonly associated with the anterior pituitary gland, but prolactin is also synthesized by the central nervous system, the immune system, mammary glands, and the uterus. Two critical roles of prolactin in lactation are promoting growth of mammary alveoli in preparation for lactation and stimulation of mammary alveolar epithelial cells to produce important components in milk [85]. To stimulate lactational protein synthesis, it acts through the Janus Kinase-Signal Transducer and Activation of Transcription (JAK-STAT) pathway [86]. Early research on the interaction between prolactin and serotonin demonstrated that the injection of serotonin into the third ventricle of the brain of male rats stimulated prolactin release [87]. Further, in lactating rats, inhibition of serotonin biosynthesis inhibited prolactin release in response to suckling stimulus [88]. At the level of the mammary gland, prolactin stimulates TPH1, thus driving serotonin synthesis [26].

Prolactin promotes epithelial cell proliferation by inducing receptor activator of NFκB ligand (RANKL), which is more notably known for its role in osteoclast differentiation and activation during bone remodeling. Once stimulated, RANKL initiates signaling in the mammary epithelial cells and participates in the terminal lobuloalveolar development during the end of pregnancy. Mice deficient in mammary RANKL or its receptor, receptor activator of NFκB (RANK), had underdeveloped mammary glands and failed to lactate [89]. Another known stimulator of RANKL is parathyroid hormone-related peptide (PTHrP), which is consequently stimulated by prolactin in the mammary gland [89, 90]. In a normal, non-diseased state, the only time PTHrP enters circulation is during lactation [91]. Parathyroid hormone-related peptide shares homology with parathyroid hormone (PTH) at the N-terminal amino acid sequence, and both act on the G-protein coupled type 1 PTH/PTHrP receptor (PTH1R) [92, 93]. During embryonic mammary gland development, PTHrP is critical for the development of the early epithelial duct system. Wysolmerski and colleagues demonstrated that when PTHrP was knocked out in all tissues but the cartilage, epithelial ducts failed to form in the mammary gland. Further, embryos that lacked either PTHrP or PTH1R displayed a failed mammary gland development [94].

### Fluoxetine During Pregnancy, Lactation, and Postpartum Depression

In 2011, 12.4% of pregnant individuals in the United States experienced a major depressive episode [95]. SSRIs are the most prescribed class of antidepressants during pregnancy and lactation and, aside from paroxetine (Paxil), are generally considered to be safe to use during this time [96]. In the United States, an estimated 13% of pregnant individuals are prescribed antidepressants, and 6% of all pregnant individuals are exposed specifically to an SSRI [14]. Of all pregnant individuals, 1.37% were exposed to fluoxetine from 2004 to 2008 [97]. Untreated depression itself can have adverse outcomes on pregnancy. Depression during pregnancy has been linked to a higher miscarriage rate, preterm delivery, low birth weights, preeclampsia, and prolonged labor [98]. Because of these risks and risks of untreated depression to the parent, many people opt to remain on SSRIs during pregnancy and lactation. However, all SSRIs are transported across the placenta; umbilical vein concentrations of fluoxetine at birth were 65% and 72% of maternal concentrations of fluoxetine and norfluoxetine, respectively [96, 99]. However, no teratogenic effects have been associated with fluoxetine exposure at any time throughout pregnancy [100, 101]. Despite the lack of evidence of teratogenic effects, there are still known risks associated with SSRI usage during pregnancy, including spontaneous abortion, preterm delivery, poor neonatal adaptation, and low birth weights, though these findings are disputed [102–104]. Interestingly, the risk of preterm and very preterm birth was decreased (16% and 50%, respectively) for people using an SSRI during pregnancy compared to untreated people with a psychiatric diagnosis [105].

Postpartum depression (PPD) affects 10 to 15% of people and can have significant consequences on both the parent and baby [13]. PPD is defined in the fifth edition of the American Psychiatric Association’s *Diagnostic and Statistical Manual of Mental Disorders* (DSM-5) as a major depressive episode that occurs during pregnancy or during the first four weeks after parturition [106]. Symptoms of PPD are similar to those of a major depressive episode and can include sleep disturbances and a lack of energy, feelings of sadness and hopelessness, anxiety, and anhedonia, or loss of interest or pleasure in normal activities. Along with the risks associated with untreated depression during pregnancy, PPD can also disrupt bonding between parent and offspring and can contribute to inadequate parental caregiving [107]. There is a relationship between depressive symptoms and issues with initiating lactation, lactation self-efficacy, and early cessation of lactation [108]. The link between PPD and lactation is not fully understood, though there is evidence supporting that breastfeeding is correlated with lower levels of depressive symptoms [109]. Breastfeeding was associated with fewer depressive symptoms, but individuals experiencing PPD are more likely to cease lactation early in the postpartum period [109]. Hatton et al. found that symptoms of depression were lower in lactating individuals at 6 but not 12 weeks postpartum, highlighting the prevalence of the inverse relationship between breastfeeding and depressive symptoms in the early postpartum period [110]. However, in vitro studies showed that drugs promoting serotonergic activity resulted in the disassembly of the tight junctions, or the barrier between epithelial cells, in the mammary gland, and fluoxetine administration to the lactating murine mammary gland was associated with changes similar to involution [111]. Additionally, people taking an SSRI during the peripartal period exhibited a greater likelihood of delayed onset of milk secretion [111].

There is evidence that points to a dysregulation of the serotonergic system early in the postpartum period. Even among people not afflicted by PPD, there is often a transient change in mood in the early postpartum period, commonly referred to as the ‘baby blues’ or the ‘postpartum blues.’ People that experience postpartum blues are at a greater risk of developing PPD [112]. Though tryptophan content is briefly raised in the plasma a few days after delivery, there is a decrease in central tryptophan due to a decrease in tryptophan transport across the blood-brain barrier a few days after birth [113]. There is also evidence that female gonadal hormones also play a role in PPD. In a study conducted by Bloch et al., non-depressed females, half of whom had a history of PPD, underwent an experiment in which the drop in estrogen and progesterone experienced after parturition was stimulated. Most people with a history of PPD had a significant mood reaction to the sudden withdrawal as opposed to the group without a history of PPD, in which none of the individuals experienced any impact on mood, suggesting that estrogen and progesterone play a role in the etiology of PPD [114].

## Calcium & Bone Metabolism

### Bone Metabolism

The skeleton is a dynamic structure that plays several important roles in vertebrate animals. Firstly, it provides protection and structural integrity of the body, as well as provides the framework for muscle, ligament, and tendon attachments, allowing for locomotion. More active roles of the skeleton include hematopoiesis and mineral storage and metabolism. There are two primary types of bones in the body: cortical and trabecular bone. Approximately 80% of the adult skeleton is cortical bone, whereas the rest is composed of trabecular bone [115]. The cortical bone is primarily responsible for stability, while the trabecular bone is more metabolically active and has a higher rate of bone turnover than cortical bone [116]. The skeleton is constantly undergoing bone remodeling, and an estimated 10% of the mature skeleton is renewed per year, with a complete renewal of the skeleton every 10 years [117, 118]. The process of bone remodeling occurs in four phases: activation, resorption, reversal, and formation [119]. Bone resorption, or bone breakdown, takes approximately 2 to 4 weeks, while the process of bone formation takes 4 to 6 months [116]. Osteoblasts, osteoclasts, and osteocytes are the three primary cell types involved in bone remodeling. Osteoblasts, which are derived from multipotent mesenchymal stem cells, are responsible for bone building. Osteoclasts, which are formed from the macrophage-monocyte cell lineage, are responsible for breaking down bone [120]. Osteocytes are mature osteoblasts that are located inside the mineralized matrix of bone as opposed to osteoblasts, which reside on the surface of bone. Specifically, osteocytes reside in the lacuna-canalicular system, which allows interstitial fluid to move through the mineralized matrix of bone [121]. Osteocytes are the most abundant type of bone cell and are long living, acting as the primary mechanoreceptors of bone by coordinating bone remodeling via communication with osteoblasts and osteoclasts [122].

Bone remodeling is a tightly regulated process, and the key signaling pathway in bone remodeling is the RANK-RANKL-OPG pathway. Receptor activator of nuclear factor kappa B (RANK) is expressed by osteoclast precursor cells. Receptor activator of nuclear factor kappa B ligand (RANKL) is produced by osteoblasts and osteocytes and promotes differentiation and activation of osteoclasts. Along with RANKL, another cytokine that is required for osteoclastogenesis is macrophage colony-stimulating factor (M-CSF), which is also produced by osteoblasts and contributes to osteoclast proliferation and differentiation [123, 124]. Osteoprotegerin (OPG) is released by osteoblasts and acts as a decoy receptor for RANKL, thus inhibiting osteoclast differentiation and activation [125].

### The Role of Serotonin in Bone Metabolism

The role of serotonin in bone remodeling was first described in 2001 when Bliziotes and colleagues demonstrated the presence of SERT and various serotonin receptors in osteoblastic cells [126]. In the same year, a separate study found that the 5-HT_2B_ receptor was expressed in fetal chick bone tissue, as well as in murine osteoblast cultures [127]. The serotonin transporter plays a significant role in serotonin-driven bone remodeling and various studies have shown that SERT is present in all major types of bone cells [126, 128, 129].

The role of gut-derived serotonin and centrally-derived serotonin in bone remodeling is widely debated and somewhat controversial, though the most prevalent theory is that gut-derived serotonin decreases bone formation, thus decreasing bone mass, and centrally-derived serotonin increases bone formation and decreases bone resorption, thus promoting bone building [130]. There are a few different mechanisms postulated for the various roles of peripheral and central serotonin in bone remodeling. One of the mechanisms in which peripheral serotonin impacts bone remodeling is through LDL-receptor related protein 5 (LPR5), which has been implicated in regulating bone remodeling. A loss-of-function mutation in LRP5 was correlated with dramatically decreased bone formation, highlighting its significance in bone metabolism. [131]. Yadav et al. reported that LRP5 exhibits its control over bone formation by inhibiting serotonin synthesis in the gut via regulation of TPH1 by using LRP5 knockout mice. These mice exhibited an increase in TPH1 expression in both the bone and the gut, an increase in circulating serotonin levels, and a low bone mass phenotype. In the same study, mice with a gut-specific TPH1 knockout had a dramatic high bone mass phenotype, and mice with an osteoblast-specific deficiency of the 5-HT_1B_ receptor also had a high bone mass phenotype [24]. Taken together, these findings suggest that gut-derived serotonin inhibits bone formation by regulating osteoblast proliferation via the 5-HT_1B_ receptor. Along with the role of gut-derived serotonin, Yadav and colleagues also proposed a mechanism in which central serotonin modulates bone remodeling. They hypothesized that brain-derived serotonin promotes bone building by decreasing sympathetic activity via the 5-HT_2C_ receptor located in the ventromedial hypothalamus. Interestingly, mice that were deficient in both TPH1 and TPH2 and thus could not synthesize serotonin anywhere in the body, showed a decrease in bone formation and an increase in bone absorption, resulting in a low bone mass phenotype [132].

Both depression and SSRIs play a role in bone remodeling. Depression itself has been linked to an increase in fracture risk and a decrease in BMD, and the severity of bone loss is correlated with the severity of depression [133, 134]. Similarly, SSRIs have been implicated in decreased bone mass and increased fracture risk [135]. Among an older population of both males and females ≥ 65 years of age, SSRI usage is associated with a decrease in BMD [134, 136]. In a population of both male and female adolescents with a mean age of 16, SSRI use for more than 6 months was associated with a lower BMD of the femur and a lower BMC of the femur and the spine [137]. Inhibition of SERT via fluoxetine administration in adult female mice resulted in a lower BMD and altered architecture, and this detrimental effect on bone was independent of estrogen deficiency [138]. Further, fluoxetine reduced osteoclast differentiation but had no impact on osteoclast activation in vitro [128]. Interestingly, Otruño et al. reported different bone phenotypes depending on the length of fluoxetine treatment. Treating C57BL6/J mice with fluoxetine for three weeks increased bone mass, whereas six weeks of treatment resulted in a net loss of bone mass. They reported that fluoxetine impaired osteoclast maturation, similar to what was reported by Battaglino. Chronic fluoxetine treatment led to decreased serotonin signaling in the brain, explaining the loss of bone mass in the context of long-term treatment [139].

### Calcium Metabolism and Bone Remodeling During Gestation

In rodents, calcium is transferred to the fetus during the last 4 to 5 days of gestation. A rat fetus will accrue calcium at a rate of less than 0.5 mg/d during the first 17 days of gestation, and this rate will increase to 12 mg/d by the end of their gestation [140]. Calcium metabolism is altered in the maternal body during gestation to accommodate the growing fetus and calcium absorption in the maternal body doubles during pregnancy. [141]. There is a multitude of factors that affect bone remodeling and mineral homeostasis in a nonpregnant, nonlactating state. An important player is 1,25-Dihydroxyvitamin D3 (1,25(OH)_2_D_3_), which promotes bone resorption by stimulating the differentiation of osteoclast precursor cells [142]. One of the physiological adaptations to the increased calcium demand during gestation is an increase in plasma 1,25-Dihydroxyvitamin D levels, but there is no increase in PTH [143, 144]. During gestation, calbindin-D9K, a calcium-binding protein that is dependent on vitamin D, is associated with increased calcium absorption [145]. 1,25-Dihydroxyvitamin D modulates calbindin-D9K to increase calcium absorption during pregnancy, but the increase in calcium absorption is not fully dependent on this mechanism; previous studies in rodents have shown an increase in calcium absorption during gestation even when 1,25-Dihydroxyvitamin D and its receptor are absent [146, 147]. In humans, the fetal skeleton accumulates approximately 30 g of calcium, and 80% of that total is accumulated during the third trimester of pregnancy [148]. The fetal skeleton will accrue calcium at a rate of 50 mg/d at 20 weeks, and this rate will increase to 330 mg/d at 35 weeks [149].

### Calcium Metabolism and Bone Remodeling During Lactation

During lactation, an average of 260 mg/L of calcium is transferred to the neonate via breast milk during the first 6 months of lactation [150, 151]. Unlike during pregnancy, calcium absorption in the intestines does not adjust for the increase in calcium demands, and instead, the maternal body sources most calcium from bone stores [151, 152]. In rodents, however, intestinal transport of calcium peaks during lactation [146]. This difference may be due, in part, to the increased calcium demand on rodents due to the increased milk requirements of a litter as opposed to one or two offspring, and the short period over which lactation occurs. To accommodate for this increase in calcium demand, the maternal skeleton will experience a 1 to 3% decrease in BMD per month, totaling a 6 to 10% loss of bone mass over the first 6 months of lactation [153]. Comparatively, rodents will lose approximately 20 to 30% of their bone mass over a 21-day lactation [153–155]. The maternal skeleton, however, does not equally contribute to calcium mobilization. In rodents, the distal femur, the proximal tibia, and the spine, all areas with the highest trabecular component of bone, have a more dramatic decrease in BMD compared to other sites that are predominantly cortical bone, such as the distal and middle tibia [155]. Interestingly, dietary calcium supplementation does not affect bone loss during lactation, nor does it impact calcium concentrations in the milk [156]. Further, dietary calcium intake did not impact the volume of milk produced or the total calcium output after 3 months of lactation [157]. The RANKL signaling pathway is critical in facilitating lactation-related bone loss. Treatment of CD1 mice with OPG completely inhibited bone resorption, preventing the rapid bone loss normally associated with lactation. Remarkably, preventing this bone loss did not affect the calcium metabolism of the dams, milk production, or milk calcium content as long as the dam was consuming sufficient dietary calcium [158].

A critical function of the mammary gland during lactation is the orchestration of calcium mobilization from the skeleton. Lactation is characterized by a decrease in estrogen due to the suppression of pulsatile gonadotropin-releasing hormone (GnRH) and the subsequently increased bone resorption due to decreased estrogen. Increased PTHrP and decreased estrogen during lactation work synergistically to increase bone turnover, accelerating both bone formation and bone resorption [159, 160]. In a lactating state, the mammary gland works in an endocrine fashion to regulate bone metabolism, allowing for calcium resorption via secretion of PTHrP from mammary epithelial cells [161]. When estrogen levels were decreased and PTHrP levels were increased in nulliparous mice, there was a marked decrease in bone mass, but the extent of bone loss during lactation was not recapitulated [162]. The mammary gland regulates its expression of PTHrP by monitoring circulating calcium via the calcium-sensing receptor (CaSR) located on mammary epithelial cells [163]. In mammary epithelial cells, CaSR and PTHrP work in a negative feedback loop to regulate calcium during lactation. Increased calcium activates CaSR, which causes a decrease in PTHrP and bone resorption and conversely, insufficient calcium downregulates CaSR, resulting in an increase in PTHrP and bone resorption [164]. PTHrP binds to PTH1R, prompting RANKL secretion from cells of the osteoblast lineage, which ultimately drives osteoclastogenesis [89]. In previous studies, mice lacking PTHrP in the mammary gland exhibited decreased calcium in milk, reduced markers of bone turnover, and partially reduced lactation-induced bone loss [163]. Interestingly, vitamin D and PTH, both calciotropic hormones that are important for bone homeostasis during a non-lactating state, were not associated with bone turnover markers or BMD changes during lactation in humans [165].

A final mechanism that participates in calcium metabolism during lactation is a process called osteocytic osteolysis. During lactation, the osteocyte lacunae enlarge at common sites of bone resorption compared to the virgin controls. Osteocytes contribute to calcium homeostasis during lactation and can mobilize bone mineral in their surrounding matrix, achieving this through PTH1R signaling by PTHrP [166, 167]. Osteocytes were shown to express markers commonly associated with osteoclast activity during lactation such as tartrate-resistant acid phosphatase (TRAP), cathepsin K, and matrix metallopeptidase 13 (MMP13) [166]. Calcitonin, a calciotropic hormone that protects the maternal skeleton from excessive breakdown during lactation, may oppose the resorptive actions of 1,25-Dihydroxyvitamin D on the skeleton by acting on its receptor to protect the bone [168]. The calcitonin receptor is expressed on osteocytes and directly acts on them to inhibit osteocytic osteolysis during lactation. Deletion of the calcitonin receptor in mice was shown to increase osteocytic osteolysis without affecting osteoclastic osteolysis [169]. Further, overexpression of calcitonin in the pituitary gland of mice resulted in a decline in circulating prolactin [170]. Because prolactin plays a role in the expression of PTHrP in the mammary gland, calcitonin may oppose the resorptive action of PTHrP via the suppression of prolactin. However, loss of calcitonin in mice was not correlated with changes in circulating prolactin during lactation compared to their calcium-replete counterparts but did show an increase in PTHrP content in the mammary tissue [171]. Therefore, calcitonin may partially counteract the resorptive effects of PTHrP-driven bone loss by acting on osteocytes to downregulate osteocytic osteolysis.

In the mammary gland, serotonin drives PTHrP secretion in an autocrine-paracrine fashion [172]. Serotonin achieves this by inducing the canonical hedgehog signaling pathway via the alteration of sonic hedgehog promoter methylation patterns, which then act to activate PTHrP synthesis in the mammary gland [173]. The hedgehog gene (Hh) was first discovered in *Drosophila* by Nusslein-Volhard and Wieschaus in 1980 [174]. Since then, three homologs of the *Drosophila* Hh gene, Sonic hedgehog (Shh), Indian hedgehog (Ihh), and Desert hedgehog (Dhh), were discovered in mice [175]. The canonical Hh signaling cascade begins when a Hh ligand binds to the Patched-1 (Ptch1) receptor, which results in the activation of Smoothened (Smo). From there, transcription of three members of the glioblastoma (Gli) family (Gli1, Gli2, Gli3) is triggered [176, 177]. Shh has two different transcription start sites (TSS), and DNA methylation of these TSS and alternative promoter usage act as transcriptional regulators of Shh expression [178, 179]. Laporta et al. were the first to describe the role of serotonin in switching TSS in the Shh promoter region to regulate PTHrP expression during lactation, further highlighting the importance of serotonin during lactation [173].

### Restoration of the Maternal Skeleton Post-Weaning

The maternal skeleton is believed to be restored after the cessation of lactation. In humans, rats, and mice, weaning triggers a change in lactation-associated bone resorption to bone-building [180–182]. Using a mouse model, Ardeshirpour and colleagues found that this restoration in bone mass is correlated with rapid cessation of bone resorption, increased osteoclast apoptosis, and a decrease in RANKL expression [180]. This post-weaning bone mass recovery has been shown to be independent of PTHrP as well, despite the requirement of osteoblast-specific PTHrP to maintain bone mass and strength in the adult. Post-weaning bone mass was recovered normally in mice with an osteoblast-specific PTHrP knockout [183]. Vitamin D plays an important role in the restoration of the maternal skeleton after weaning. Bone demineralization during lactation occurs in the absence of vitamin D in rodents. However, bone loss during lactation is not restored in vitamin D deficient rats as opposed to their vitamin D replete counterparts [146].

There is a considerable amount of evidence from rodent studies that bone mass is completely restored after the cessation of lactation. However, it is important to note the physiological changes in lactation strategies and calcium metabolism during lactation between rodents and humans. Rodents are a litter-bearing species and experience an intense milk demand over a short period. They also have a far shorter lifespan than humans and do not experience menopause. Therefore, it is important to turn to epidemiological data in humans to further explore whether bone mass is fully restored after lactation and what factors may impact post-weaning bone recovery. There are conflicting conclusions in the literature on whether bone mass is fully recovered after lactation. Many studies have reported that bone mass is fully restored at most or all of the primary sites of lactation-driven bone resorption in most women post-weaning [184–188]. Many epidemiologic studies have also found that there was either a neutral or protective effect of lactation in factors such as BMD and fracture risk in both premenopausal and postmenopausal women [189–194]. However, some studies suggest that lactation has a negative impact on bone mass and that this negative impact might worsen with extended lactation [195, 196]. There is also evidence that lactation also increases fracture risk in postmenopausal women and that the risk might increase with extended lactation [197]. In epidemiologic studies, other factors may explain the discrepancies between studies, such as dietary influence, lifestyle choices, and the differences between populations of people that participated in these studies.

## Early Development and Fetal Programming

### Skeletal Development

The skeleton is a complex structure formed of cartilage and bone and is produced by three embryonic lineages during development. Cranial neural crest cells form the craniofacial skeleton, the paraxial mesoderm, or somites, forms the axial skeleton, and the lateral plate mesoderm forms the appendicular skeleton [198]. The axial skeleton includes the skull, spine, sternum, and ribs, and the appendicular skeleton includes the bones of the limbs. There are two primary processes in which bone develops in vertebrates: intramembranous or endochondral ossification. Endochondral ossification is the process in which a cartilage template is replaced by bone, and intramembranous ossification is the mineralization of the mesenchyme via direct osteoblast differentiation [199]. Parts of the skull, the axial skeleton, and the appendicular skeleton are formed by endochondral ossification, while the flat bones, which include the scapula, sternum, and cranium, are formed by intramembranous ossification [200]. In humans, bone formation begins in weeks 4 to 5 of gestation with the formation of mesenchymal cell clusters [201]. The skeleton grows rapidly in fetuses and infants, slows down during childhood, and then grows rapidly again during puberty. Growth then continues until, generally, the end of the second decade [202].

The process of endochondral ossification is tightly regulated and begins in the fetus, continuing postnatally until the skeleton reaches full maturity. Ossification begins when mesenchymal cells are derived from the mesoderm condensate. The cells toward the center of the condensations differentiate into chondrocytes and begin to produce an extracellular matrix, which includes factors such as type II collagen and aggrecan. This will eventually form a cartilage template for bone formation. The mesenchymal cells toward the outside of the condensations develop into the perichondrium. Appositional growth is dictated by precursor cells of the perichondrium, while longitudinal growth occurs via interstitial chondrocyte division. Once a certain size is achieved and the cartilage template is formed, the chondrocytes within the cartilage will begin to hypertrophy, which triggers ossification within the perichondrium [203]. During chondrocyte hypertrophy, blood vessels innervate the cartilage template and deliver osteoblasts and osteoclasts, which ultimately form a structure called the primary ossification center. Concurrently, osteoblast differentiation occurs within the perichondrium, which results in the secretion of type I collagen and the formation of the bone collar [204]. In the long bones, a secondary ossification center forms at the end of the cartilage template and forms the epiphyseal plate, or growth plate, which is responsible for longitudinal growth that persists postnatally [205]. In humans, this longitudinal growth continues until chondrocyte proliferation declines and the growth plate experiences epiphysial fusion at the end of puberty [206, 207]. Rodents do not undergo epiphyseal fusion until much later in life, far past sexual maturity [208].

PTHrP is an essential regulatory factor during endochondral ossification. Before chondrocyte maturation, PTHrP is expressed in the perichondrium and early chondrocytes, and its receptor is expressed in early chondrocytes. After chondrocyte maturation, PTHrP is expressed in the perichondrium and hypertrophic chondrocytes located at the ends of bones [209]. During development, PTHrP acts on the PTH1R receptor of nearby chondrocytes [210]. The PTH1R receptor is minimally expressed in proliferating chondrocytes and is highly expressed in prehypertrophic chondrocytes, and PTHrP acts on it to delay chondrocyte hypertrophy and promote chondrocyte proliferation. In the absence of PTHrP, chondrocytes undergo differentiation. Indian hedgehog regulates PTHrP expression in the developing skeleton via the canonical Hh signaling pathway and is expressed by prehypertrophic chondrocytes [211]. To regulate chondrocyte differentiation, a negative feedback loop between Ihh and PTHrP is established in the developing skeleton [212]. Prehypertrophic chondrocytes produce Ihh which induces PTHrP expression thereby preventing chondrocytes from differentiating. Preventing chondrocytes from becoming prehypertrophic thus suppresses the expression of Ihh. In this way, Ihh and PTHrP form a negative feedback loop to modulate chondrocyte differentiation and maintenance of the growth plate. In 1999, St-Jacques and colleagues demonstrated that Ihh was essential for bone formation. Mice without Ihh expression had reduced chondrocyte proliferation, delayed and abnormal chondrocyte maturation, and the absence of mature osteoblasts in endochondral bones [213]. Conversely, PTHrP null mice exhibited severely reduced growth plate size in the long bones and premature chondrocyte proliferation, resulting in premature bone formation [214].

Maturation of osteoblasts from mesenchymal stem cells requires the expression of transcription factors such as runt-related transcription factor 2 (Runx2), also known as core-binding factor subunit α-1 (CBFα1), and Osterix (Osx), which acts downstream of Runx2 [215, 216]. Thus, Runx2 is essential for both intramembranous and endochondral ossification [215]. Mice lacking Runx2 displayed a complete lack of both intramembranous and endochondral ossification due to the lack of osteoblast maturation [215]. In chondrocytes, osteoblast progenitor cells, and mature osteoblasts, Runx2 regulates Ihh expression [217, 218]. Another important component in osteoblast and chondrocyte differentiation is the Wingless (Wnt)/β-catenin signaling pathway, which acts synergistically with the Ihh signaling pathway in endochondral bone formation [219, 220]. During development, Wnt/β-catenin signaling antagonizes PTHrP signaling to regulate chondrocyte hypertrophy, though Wnt/β-catenin control of the final maturation of hypertrophic chondrocytes works independently of PTHrP [221].

### Fluoxetine During the Peripartal Period and Neonatal Outcomes

There is an interplay between the placenta, the embryo, and the mother in terms of the source of serotonin in early pregnancy. Serotonin, a variety of serotonin receptors, and SERT are present in preimplantation embryos [222–224]. During early development, maternally-sourced serotonin is critical for proper embryonic development. The pups of TPH1-null dams showed gross developmental abnormalities regardless of whether they were TPH1^+/−^ or TPH1^−/−^, suggesting that maternal serotonin and not fetal serotonin is critical for early fetal development [225]. Along with maternally-sourced serotonin, the placenta also proves to be a source of serotonin to the fetus during development. The placenta can synthesize its own serotonin from maternal tryptophan starting at E10.5 in the mouse and 11 weeks of gestation in humans [226, 227]. In the periphery of the mouse fetus, endogenous serotonin is detected at E16 in the gut, hinting at the change from placental to fetal sources of serotonin during development [228]. In rodent studies, there is evidence that fluoxetine has an impact on litter size and pup mortality in a dose-dependent manner. Vorhees and colleagues demonstrated that, when treated on days 7–20 of pregnancy, rats exposed to fluoxetine experienced maternal weight loss, smaller litters, and higher rates of pup mortality at a 12 mg/kg dose, which represents the upper level of a relative therapeutic dose in humans, but not at a 5 mg/kg dose [229]. Despite the deleterious perinatal effects, there was no effect on growth or survival in the long-term at the high dose [230]. In a separate study, when rats were administered fluoxetine on days 6 to 20 of pregnancy, rats exposed to fluoxetine developmentally exhibited a decreased birth weight and a slowed weight gain pre-weaning at a 12 mg/kg dose, but not at an 8 mg/kg dose [231]. In a study conducted in sheep, which are more physiologically relevant to humans than rodents, pregnant ewes were administered 50 mg of fluoxetine for 14 days at the end of pregnancy. Mean gestation length and birth weight of lambs treated with fluoxetine did not differ from untreated controls. Interestingly, fluoxetine-treated lambs took less time to stand, walk, and suckle than untreated controls [232].

A variety of conclusions have been made based on studies of fluoxetine exposure and neonatal outcomes in humans. In a study of 228 pregnant women between 1989 and 1995, fluoxetine usage during late pregnancy resulted in an increased rate of prematurity and poor neonatal adaptation [102]. Along with those findings, mean birth weight and gestation length of infants exposed to fluoxetine only during late pregnancy were less than that of infants exposed only during early pregnancy [102]. In a study of 64 infants, gestational age, birth weight, or Apgar scores did not differ between infants exposed to fluoxetine during early versus late pregnancy [103]. Neither of these studies controlled for the degree of depression in the mother or the concurrent use of other psychiatric medications. Suri et al. conducted a study of 64 women between 1997 and 2000 which excluded women that were on other psychiatric medication or using tobacco, alcohol, or other substances. No differences were found in gestational age, birth weight, or Apgar scores in the infants of depressed women treated with fluoxetine, untreated depressed women, and the control group [233]. None of these studies took the dosage into account, which might act as a confounding factor given the dose-dependent response demonstrated in animal studies. A more recent study conducted on 145 women between 2015 and 2018 found that, when controlling for symptoms of depression and anxiety in the mother, a higher antidepressant dosage was significantly correlated with lower birth weight, but not length of gestation [234]. However, this study did not differentiate between individual antidepressants and included results from both the SSRI and the serotonin norepinephrine reuptake inhibitor (SNRI) classes of antidepressants; in this study, only 12 of the participants were on fluoxetine [234]. There is a considerable amount of variability in human studies regarding fluoxetine usage during pregnancy and birth outcomes, but there is evidence that there may be a link between it and adverse neonatal outcomes in human pregnancies. In a study performed on sheep, fluoxetine administration during late gestation resulted in a transient decrease in uterine artery blood flow, which can be associated with fetal hypoxemia and other detrimental respiratory outcomes. However, interestingly, this transient decrease did not cause any negative birth outcomes such as birth weight, length of gestation, or intrauterine growth restriction [235].

While considering the impact of fluoxetine on neonatal outcomes, it is important to reiterate the risks of untreated depression during gestation. Ranzil et al. studied human placentas from both normal pregnancies and FGR pregnancies and found that TPH2 mRNA was decreased in the FGR placentas compared to the normal placentas and that there was also enhanced activity of both TPH1 and TPH2 enzymes in the FGR placentas [236]. People with a depression diagnosis were found to be 1.2 to 2.8 times more likely to experience unfavorable maternal and/or fetal outcomes, and there is a positive association between depression and preterm labor and fetal growth restriction (FGR) [237, 238]. Further, depression during pregnancy was also associated with fetal distress and abnormalities [237, 239]. This highlights the potential role of serotonin in normal pregnancies and the possible mechanism by which serotonin perturbation, whether pharmacologically or psychiatrically induced, can lead to adverse neonatal outcomes.

### ***In Utero*** and Lactational Exposure to Fluoxetine

Fluoxetine and norfluoxetine pass through the placenta and are present in breastmilk [240]. Infant exposure to both fluoxetine and norfluoxetine via breastmilk was 2.4% of the maternal weight-adjusted daily dose at 2 weeks of age and 3.8% at 2 months of age [99]. There is conflicting evidence on whether fluoxetine exposure is associated with teratogenic effects in the offspring. Most SSRIs sit in this gray area except for paroxetine (Paxil), which has an established link with congenital malformations and major cardiac malformations [241]. Sex hormones have an established relationship with serotonin. Because of this, the impact of fluoxetine exposure has been investigated in terms of sex differences in the exposed offspring. Both *in utero* and lactational exposure to fluoxetine resulted in a delayed onset of puberty in female rat offspring but had no effect on the estrus cycle in adult female mice [242]. Dos Santos and colleagues included the caveat, however, that the onset of puberty was examined only 9 days after the end of fluoxetine exposure, while the estrus cycle was examined 54 days after the end of fluoxetine exposure, which may have had an influence on the results.

Much like SSRI usage in adults and the subsequent impact on bone health, SSRIs may have an impact on bone during development. In a study of 80 newborn infants, infants exposed to SSRI had a smaller head circumference than non-exposed infants, but SSRI usage during gestation was not correlated with a decrease in BMD. However, it is important to note that this study used quantitative ultrasound instead of dual-energy X-ray absorptiometry (DEXA), which is the gold standard in human medicine for measuring BMD. Along with that limitation, the study included a variety of SSRI medications at different doses [243]. Inhibition of SERT via both SERT-null mice and fluoxetine administration resulted in a phenotype of decreased bone mass and altered bone architecture [244, p2]. In both models, SERT inhibition resulted in a reduction in bone accrual [244]. When rat dams were orally dosed with 20 mg/kg, their offspring displayed differences in the mandibular bone, including reduction of osteocyte number and reduction in bone density [245]. However, it is important to note that most of the mandible is formed via intramembranous ossification, which is a different process than endochondral ossification [246].

## Conclusions

The lactating mammary gland orchestrates calcium homeostasis via alterations of bone metabolism through a serotonin-dependent mechanism. Further, serotonin plays a critical role in fetal and neonatal bone development. Disruptions in the serotonergic system of the peripartal mother or the developing offspring can potentially have lasting impacts on the skeleton. Fluoxetine was first introduced into the United States in 1987, and so the first possible cohort of adults that took fluoxetine during pregnancy and lactation are now reaching an age in which menopause typically occurs. Additionally, the adults that were developmentally exposed to fluoxetine *in utero* or during lactation are now reaching their mid-thirties, a time frame just after peak bone mass is established in humans. Therefore, it is critical to assess perturbations in the serotonergic system via fluoxetine on the bones of both the mother and offspring to assess the potential long-term effects of fluoxetine treatment.
